# Integrative Transcriptome and Proteome Analysis Reveals the Absorption and Metabolism of Selenium in Tea Plants [*Camellia sinensis* (L.) O. Kuntze]

**DOI:** 10.3389/fpls.2022.848349

**Published:** 2022-02-24

**Authors:** Hengze Ren, Xiaoman Li, Lina Guo, Lu Wang, Xinyuan Hao, Jianming Zeng

**Affiliations:** ^1^National Center for Tea Improvement, Key Laboratory of Tea Biology and Resources Utilization, Ministry of Agriculture and Rural Affairs, Tea Research Institute, Chinese Academy of Agricultural Sciences, Hangzhou, China; ^2^College of Horticulture, Fujian Agriculture and Forestry University, Fuzhou, China

**Keywords:** *Camellia sinensis*, transcriptome, proteome, selenite, selenate

## Abstract

Certain tea plants (*Camellia sinensis*) have the ability to accumulate selenium. In plants, the predominant forms of bioavailable Se are selenite (SeO_3_^2–^) and selenate (SeO_4_^2–^). We applied transcriptomics and proteomics to hydroponically grown plants treated with selenite or selenate for 48 h in the attempt to elucidate the selenium absorption and assimilation mechanisms in tea. A total of 1,844 differentially expressed genes (DEGs) and 691 differentially expressed proteins (DEPs) were obtained by comparing the Na_2_SeO_3_ and Na_2_SeO_4_ treatments against the control. A GO analysis showed that the genes related to amino acid and protein metabolism and redox reaction were strongly upregulated in the plants under the Na_2_SeO_3_ treatment. A KEGG pathway analysis revealed that numerous genes involved in amino acid and glutathione metabolism were upregulated, genes and proteins associated with glutathione metabolism and ubiquinone and terpenoid-quinone biosynthesis were highly expressed. Genes participating in DNA and RNA metabolism were identified and proteins related to glutathione metabolism were detected in tea plants supplemented with Na_2_SeO_4_. ABC, nitrate and sugar transporter genes were differentially expressed in response to selenite and selenate. Phosphate transporter (*PHT3;1a*, *PHT1;3b*, and *PHT1;8*) and aquaporin (*NIP2;1*) genes were upregulated in the presence of selenite. Sulfate transporter (*SULTR1;1* and *SULTR2;1*) expression increased in response to selenate exposure. The results of the present study have clarified Se absorption and metabolism in tea plants, and play an important theoretical reference significance for the breeding and cultivation of selenium-enriched tea varieties.

## Introduction

Selenium (Se) is a trace non-metal element that is sometimes regarded as a metalloid. It is an essential mineral nutrient for humans, animals, and certain microorganisms. At the appropriate concentrations, it is beneficial for plant growth (Guignardi and Schiavon, 2017). Selenium may be toxic or beneficial depending on the dosage. High Se concentrations are phytotoxic as they cause oxidative stress. Moreover, they form selenoamino acids that interfere with protein folding and function (Guignardi and Schiavon, 2017). At optimal doses, Se protects plants against oxidative stress, reduces the toxicity of harmful elements, regulates growth, photosynthesis, respiration, and improves yield and quality (Hu et al., 2003; Feng et al., 2013; Yu et al., 2019; Chauhan et al., 2020; Niu et al., 2020). In humans, optimal Se doses improve antioxidant capacity, prevent cancer, and reduce heavy metal toxicity (Pieczyñska and Grajeta, 2015). However, global Se distribution is highly heterogeneous. Over 15% of the world’s population suffers from Se deficiency characterized by Kashin–Beck and Keshan diseases (Tan et al., 2016). Tea [*Camellia sinensis* (L.) O. Kuntze] is an economically important perennial woody plant grown in many Asian, African, and Latin American countries. It is used to prepare a non-alcoholic beverage consumed by over three billion people in 160 countries (Xia et al., 2017; Wang et al., 2020). Certain tea cultivars can accumulate selenium. Within the plant, 80% of the Se occurs as moieties of organic compounds that can be absorbed through the human digestive tract (Gao et al., 2014). In regions where the soil Se content is low, the amount of Se in tea is actually greater than the available Se in maize, rice, beans, or potatoes (Xiang et al., 2012). Thus, tea may be an ideal alternative Se supplement for humans. However, little is known about the mechanisms by which tea plants absorb, translocate, or metabolize Se.

In nature, the existence valence of Se mainly includes −2 in selenide, 0 in elemental selenium, +2 in thioselenate, +4 in selenites, +6 in selenates. The predominant inorganic forms of Se available to plants are selenate in oxic soils (pE + pH > 15) and selenite in anaerobic soils (7.5 < pE + pH < 15) (Sors et al., 2005; White, 2016). Plants absorb selenate and selenite via sulfate and phosphate transporters, respectively. Tea and other plants have four different types of sulfate transporters (Gigolashvili and Kopriva, 2014; Zhang et al., 2021). In *Arabidopsis thaliana*, *SULTR1;1* and *SULTR1;2* are high-affinity sulfate transporters that absorb selenate. *SULTR2;1* and *SULTR2;2* are low-affinity sulfate transporters that mediate selenate translocation from the roots to the leaves (Takahashi et al., 2000; El Kassis et al., 2007; Takahashi, 2019). *CsSULTR1;2/2;1/3;3/3;5* were upregulated in response to Se^4+^ and Se^6+^ exposure in *Camellia sinensis* (Zhang et al., 2021). Plant phosphate transporters are classified into subfamilies PHT1–5 (Liu et al., 2016; Wang et al., 2017). Selenite uptake is mediated by phosphate transporters (Zhang et al., 2014; Song et al., 2017). Only PHT1, PHT3, PHT4, and PHO were identified in *C. sinensis* (Cao et al., 2021). A transcriptome analysis of tea plants treated with selenite showed that their phosphate transporters may participate in selenite absorption and translocation (Cao et al., 2021). Nevertheless, the phosphate transporters involved in the uptake and translocation of Se species with different valences remain to be further elucidated.

After selenate is absorbed by the roots, it can be reduced to selenite via ATP sulfurylase and APS reductase. The selenite is then reduced to Se^2–^ (selenide) either enzymatically by sulfite reductase or non-enzymatically via glutathione. Cysteine and methionine synthases may then catalyze the formation of the selenoamino acids selenocysteine and selenomethionine, respectively (Guignardi and Schiavon, 2017). Few studies to date have been conducted on the metabolism of Se with different valences in tea plants.

Transcriptomics, metabolomics, and proteomics have been used to study Se disposition in living organisms (Zhang C. et al., 2019; Chauhan et al., 2020; Rao et al., 2021). Accordingly, multiple omics technologies should also be applied to explore Se uptake and metabolism in tea plants. In the present study, we integrated transcriptomics and proteomics to clarify the mechanisms of selenite and selenate uptake and metabolism in tea plants. We believe that the results of this research will provide theoretical guidance for breeding and cultivating Se-accumulating tea varieties.

## Materials and Methods

### Plant Materials and Treatments

One-year-old tea plant cuttings (*C. sinensis* cv. ‘Zhongcha 108’) were placed in black containers holding 1/4 nutrient solution (pH 5.0) consisting of ammonium salt (80.04 mg/L), KH_2_PO_4_ (8.53 mg/L), K_2_SO_4_ (52.27 mg/L), MgSO_4_ (80.64 mg/L), CaCl_2_ (58.82 mg/L), Al_2_(SO_4_)_3_ (23.33 mg/L), EDTA-FeNa (1.77 mg/L), H_3_BO_3_ (0.43 mg/L), MnSO_4_⋅H_2_O (0.17 mg/L), ZnSO_4_⋅7H_2_O (0.19 mg/L), CuSO_4_⋅5H_2_O (0.03 mg/L), and (NH_4_)_2_MoO_4_ (0.06 mg/L) (Ruan et al., 2007). The solution was renewed once weekly. The plants were grown in a greenhouse under a 12-h photoperiod and at 22°C and 70% RH. After the adventitious roots emerged, the cuttings were transferred to fresh 1/4 nutrient solution without Se or supplemented with 5 μM Na_2_SeO_3_ or 5 μM Na_2_SeO_4_. Each treatment consisted of four pots and each pot held 40 cuttings. After 48 h, roots and young shoots (two leaves and one bud) were sampled for total Se content determination. Other roots were promptly frozen in liquid nitrogen and stored at −80°C until the transcriptomic and proteomic analyses. Four biological replicates were conducted per analysis.

### Se Content Determination

The roots were rinsed with MilliQ water containing 2 mM MES and 1 mM CaSO_4_ and dried with absorbent paper. The roots and shoots were freeze-dried for 24 h in a lyophilizer (TF-FD-1; Zhejiang Nade Scientific Instrument Co. Ltd., Hangzhou, China). The samples were then pulverized for total Se content determination according to a previously described method (Zhang H. et al., 2019).

### RNA Isolation, Library Construction, and Illumina Sequencing

Total RNA was isolated from the roots according to a previously described method (Chang et al., 1993). The integrity of the total RNA was assessed with the RNA Nano 6000 Assay Kit and the Bioanalyzer 2100 System (Agilent Technologies, Santa Clara, CA, United States). The library was prepared with a NEB Next Ultra RNA Library Prep Kit (New England Biolabs, Ipswich, MA, United States) according to a previously described method (Parkhomchuk et al., 2009) and sequenced on an Illumina Novaseq platform (Illumina, San Diego, CA, United States).

### Transcriptomic Data Analysis

Clean reads were obtained by removing low-quality reads and those with adapter sequences from the raw data. They were then mapped to the reference genome with HISAT2 v. 2.0.5^1^. Novel genes were identified with StringTie v. 1.3.3b^2^. The expected numbers of fragments per kilobase of transcript sequence per million base pairs sequenced (FPKM) were calculated with FeatureCounts v. 1.5.0-p3^3^. Differentially expressed genes (DEGs) were filtered with padj < 0.05 and | log_2_ (FoldChange)| > 0.5 using DESeq2 v. 1.20.0^4^ (Love et al., 2014). Gene ontology (GO) and Kyoto Encyclopedia of Genes and Genomes (KEGG) enrichment analyses were performed on all DEGs as previously described (Wang L. et al., 2019).

### Gene Expression via RT-qPCR

One hundred milligrams tea roots was used for total RNA extraction in the RNAprep Pure Plant Plus Kit [Tiangen Biotech (Beijing) Co. Ltd., Beijing, China]. RNA samples (1 mg) were then treated with RNase-free DNase I (TaKaRa Bio Inc., Kusatsu, Japan) to remove residual genomic DNA. The cDNA was then synthesized with a PrimerScript RT Reagent Kit (TaKaRa Bio Inc., Kusatsu, Japan) according to the manufacturer’s protocol. The cDNA products were diluted tenfold and used as a PCR template. The RT-qPCR was conducted as previously described (Hao et al., 2018). The *CsPTB* reference gene was the internal control (Hao et al., 2014).

### Proteome Profiling

The protein was extracted and quantified as previously described (Wu et al., 2014), labeled with TMT labeling reagent, mixed in equal volumes, desalted, and lyophilized (Zhang et al., 2016). The powder was then dissolved in 2% (v/v) acetonitrile (pH 10.0) and centrifuged at 12,000 × *g* and 25°C for 10 min. The samples were then fractionated in a C18 column (Waters BEH C18; 4.6 mm × 250 mm; 5 μm; Waters Corp., Milford, MA, United States) on a Rigol L3000 HPLC system (Arc Scientific, Boston, MA, United States). The eluates were measured at 214 nm, collected every minute, and combined into ten fractions. The latter were dried under vacuum and reconstituted in 0.1% (v/v) formic acid. A 1-μg sample was analyzed by ultra-high performance liquid chromatography (EASY-nLC 1200 UHPLC, Thermo Fisher Scientific, Waltham, MA, United States) coup™led with tandem mass spectrometry (Q Exactive™ HF-X, Thermo Fisher Scientific, Waltham, MA, United States). The proteome profiling results were presented in Supplementary Tables 1, 2.

### Proteomic Data Analysis

Each spectrum was separately searched with Proteome Discoverer v. 2.4 (PD 2.4; Thermo Fisher Scientific, Waltham, MA, United States). To improve output quality, the results were filtered with PD 2.4. Data with credibility >99% were identified as Peptide Spectrum Matches (PSMs). Each identified protein contained at least one unique peptide. The PSMs and protein were retained and analyzed using a false discovery rate (FDR) ≤ 1.0%. Protein quantitation was statistically analyzed with a *t*-test. Differentially expressed proteins (DEPs) were identified as those with *P* < 0.05. A GO functional analysis was conducted using an interproscan program against the non-redundant protein databases Pfam^5^, PRINTS^6^, ProDom^7^, SMART^8^, ProSite^9^, and PANTHER^10^ (Jones et al., 2014). KEGG (Kyoto Encyclopedia of Genes and Genomes) was used to analyze the protein pathway.

### Transcriptome and Proteome Correlation Analyses

Alignment analyses of the gene sequences identified from the transcriptome and protein sequences were conducted at https://magic.novogene.com. The DEG and DEP data were integrated, GO enrichment and KEGG pathway analyses were performed, and the results were displayed in a heatmap.

### Statistical Analysis

Transcriptomic sequencing, proteomic analysis, and RT-qPCR were conducted in four biological replicates. Statistical analyses were run in SPSS Statistics v. 19 (IBM Corp., Armonk, NY, United States) and consisted of ANOVA followed by the LSD test at *P* < 0.05. Column plots were drawn with GraphPad Prism 8 (GraphPad Software, La Jolla, CA, United States). Heatmaps were plotted with Tbtools^11^ (Chen et al., 2020). Adobe Photoshop CS5^12^ was used to assemble the figures.

## Results

### Effects of Selenite and Selenate Exposure on Root and Leaf Se Content

**FIGURE 1:**
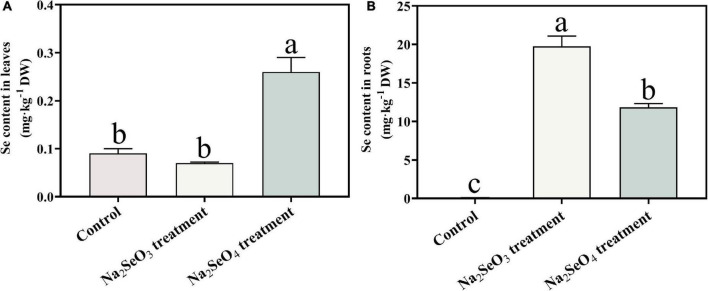
Total Se content in leaves **(A)** and roots **(B)** of tea plants treated with selenite and selenate. Different lowercase letters in the figure indicate the significant difference at *P* < 0.05 level.

Total Se was measured in the roots and leaves of tea plants subjected to Na_2_SeO_3_ or Na_2_SeO_4_ for 48 h. Samples without selenium supplementation served as the control. The total Se content in the roots significantly (*P* < 0.05) increased in response to Na_2_SeO_3_ treatment (Figure 1). The tea plants absorbed selenite more effectively than selenate. The total Se content in the leaves significantly (*P* < 0.05) increased in response to Na_2_SeO_4_ treatment. However, there was no significant difference between the Na_2_SeO_3_ treatment and the control in terms of foliar Se content (Figure 1). Hence, selenate was relatively more efficiently transported than selenite from the roots to the leaves.

### Differentially Expressed Genes and Proteins in Response to Selenite and Selenate

**FIGURE 2:**
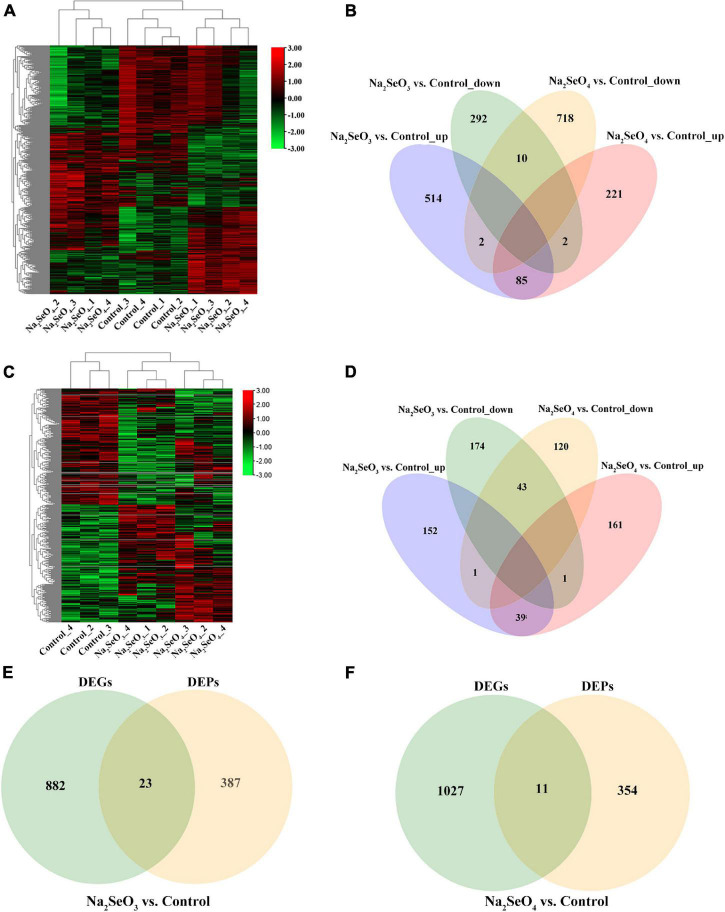
Heatmap and Venn diagram analyses of differentially expressed genes (DEGs) and differentially expressed proteins (DEPs) in response to selenite and selenate treatments. **(A)** DEG heatmap. **(B)** Venn diagram showing overlap in numbers of upregulated and downregulated DEGs between Na_2_SeO_3_ vs. control and Na_2_SeO_4_ vs. control. **(C)** DEP heatmap. **(D)** Venn diagram showing overlap in numbers of upregulated and downregulated DEPs between Na_2_SeO_3_ vs. control and Na_2_SeO_4_ vs. control. **(E,F)** Venn diagram showing overlap in numbers of DEGs and DEPs for Na_2_SeO_3_ vs. control and for Na_2_SeO_4_ vs. control, respectively.

To elucidate the Se absorption and metabolism mechanisms in tea plants exposed to selenite and selenate, we compared relative gene and protein expression in untreated hydroponic ZC108 and in those subjected to 5 μM Na_2_SeO_3_ or 5 μM Na_2_SeO_4_ for 48 h. RNA-seq revealed 532.02 million clean reads (79.80 Gb) after data filtering and quality evaluation. The reads ranged in size from 5.98 to 7.16 Gb per sample (Supplementary Table 3). A heatmap analysis showed 2,272 DEGs based on comparisons of the three transcriptional datasets (padj < 0.05; | log_2_(FoldChange)| > 0.5) (Figure 2A). Venn analyses revealed 806 and 939 unique genes for Na_2_SeO_3_ vs. control and Na_2_SeO_4_ vs. control, respectively. However, 99 genes were common to both groups (Figure 2B). In addition, 601 and 308 DEGs were upregulated while 730 and 304 DEGs were downregulated for Na_2_SeO_3_ vs. control and Na_2_SeO_4_ vs. control, respectively (Figure 2B).

The TMT generated 41,038 peptides and 6,632 proteins. Of the latter, 6,596 were quantified (Supplementary Table 4). Moreover, 192 and 201 DEPs were upregulated while 218 and 164 DEPs were downregulated for Na_2_SeO_3_ vs. control and Na_2_SeO_4_ vs. control, respectively (*P* < 0.05) (Figures 2C,D).

We conducted correlation analyses of the DEGs and DEPs to disclose mutual regulation between genes and proteins in response to selenite and selenate. For Na_2_SeO_3_ vs. control and Na_2_SeO_4_ vs. control, 23 and 11 DEPs and their corresponding DEGs were identified, respectively (Figures 2E,F).

### Differentially Expressed Genes and Differentially Expressed Proteins Gene Ontology Enrichment and Kyoto Encyclopedia of Genes and Genomes Pathway Analyses

**FIGURE 3:**
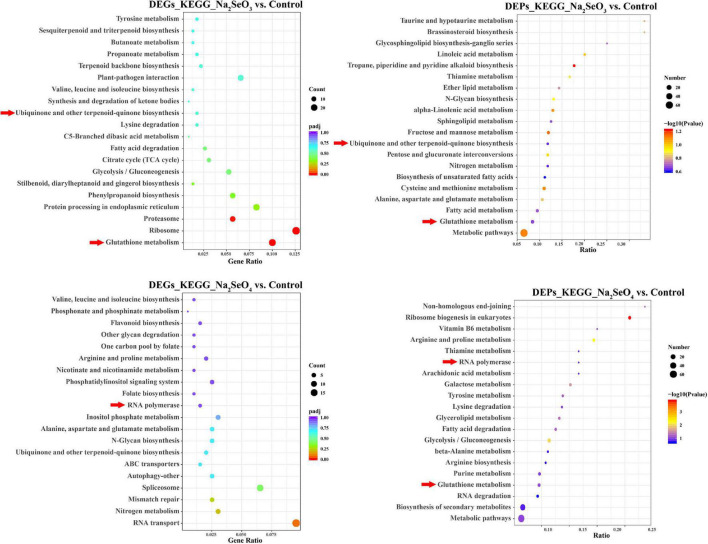
Kyoto Encyclopedia of Genes and Genomes (KEGG) pathway analysis of DEGs and DEPs in response to selenite and selenate treatment, respectively.

We subjected the unique 905 and 1,038 DEGs separately identified for Na_2_SeO_3_ vs. control and Na_2_SeO_4_ vs. control to GO enrichment and KEGG pathway analyses. For Na_2_SeO_3_ vs. control, DEGs related to protein metabolism and redox reaction were highly enriched. GO terms such as ‘cellular protein catabolic process,’ ‘proteolysis involved in cellular protein catabolic process,’ ‘protein catabolic process,’ and ‘peptide biosynthetic process’ under biological process, ‘proteasome core complex,’ ‘peptidase complex,’ and ‘ribonucleoprotein complex’ under cellular component, and ‘oxidoreductase activity acting as donors,’ ‘oxidoreductase activity acting as donors and acceptor,’ and ‘oxidoreductase activity acting on paired donors’ under molecular function were enriched (Supplementary Figure 1). For the KEGG pathway analysis, ‘glutathione metabolism,’ ‘ribosome,’ and ‘proteasome’ were highly enriched (Figure 3). For Na_2_SeO_4_ vs. control, four of the top ten GO terms under biological process were related to RNA and DNA metabolism. These included ‘mRNA metabolic process,’ ‘RNA processing,’ ‘DNA repair,’ and ‘cellular response to DNA damage stimulus.’ Under cellular component, the GO terms were related mainly to nuclear, plasma membrane, and organellar processes. Under molecular function, ADP binding was significantly enriched (Supplementary Figure 1). For the KEGG pathway analysis, ‘RNA transport,’ ‘nitrogen metabolism,’ and ‘mismatch repair’ were highly enriched (Figure 3). The DEPs were also subjected to KEGG pathway analysis. ‘Metabolic pathways,’ ‘glutathione metabolism,’ and ‘fatty acid metabolism’ were highly enriched for Na_2_SeO_3_ vs. control while ‘metabolic pathways,’ ‘biosynthesis of secondary metabolites,’ and ‘RNA degradation’ were highly enriched for Na_2_SeO_4_ vs. control (Figure 3). Among the top twenty KEGG pathways, ‘glutathione metabolism’ and ‘tyrosine metabolism’ were enriched in the transcriptomic and proteomic analyses of Na_2_SeO_3_ vs. control while ‘RNA polymerase’ was enriched in the transcriptomic and proteomic analyses of Na_2_SeO_4_ vs. control (Figure 3).

### Differentially Expressed Genes Involved in Putative Se Transport and Assimilation

**FIGURE 4:**
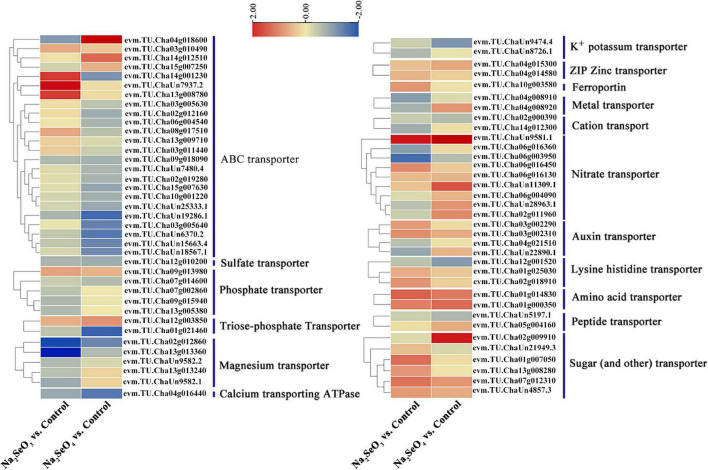
Heatmap of putative Se transporters identified from DEGs. Different colors indicate different gene expression levels based on log_2_ FoldChange. The same below.

The transcriptome revealed 1,119 transporters. Of these, 85 were differentially expressed in response to selenite and selenate treatment (padj < 0.05; |log_2_(FoldChange)| > 0.5). Seventy-three genes were categorized into calcium-transporting ATPase and the following transporters: ABC, sulfate, phosphate, triose-phosphate, magnesium, potassium, ZIP zinc, ferroportin, metal, cation, nitrate, auxin, lysine histidine, amino acid, peptide, sugar (and other), and others (Figure 4). For Na_2_SeO_3_ vs. control, 24 ABC transporter genes were identified. Three (evm.TU.Cha14g001230, evm.TU.ChaUn7937.2, and evm.TU.Cha13g008780) were upregulated 3. 60-, 5. 23-, and 3.63-fold, respectively, while 13 were downregulated. Of the nine nitrate transporter DEGs, four were upregulated and the expression levels of evm.TU.ChaUn9581.1 and evm.TU.Cha06g016450 had increased 4.79- and 2.01-fold, respectively. Of the eight sugar (and other) transporter DEGs, six were upregulated and the expression levels of evm.TU.Cha07g012310 and evm.TU.Cha01g007050 had increased by 2.41- and 2.51-fold, respectively. For Na_2_SeO_4_ vs. control, evm.TU.Cha04g018600 and evm.TU.Cha14g012510 of the ABC transporter were upregulated by 7.16- and 2.58-fold, respectively, and 18 genes were downregulated. Eight of the nitrate transporters genes were upregulated. Of these, evm.TU.ChaUn9581.1, evm.TU.ChaUn11309.1, and evm.TU.Cha02g011960 were upregulated by 7. 93-, 3. 04-, and 2.00-fold, respectively. Six sugar (and other) transporter genes were upregulated and evm.TU.Cha02g009910 was upregulated by 4.64-fold (Supplementary Table 5). Hence, certain genes regulating the ABC, nitrate, and sugar transporters might also control selenite and selenate uptake and allocation.

**FIGURE 5:**
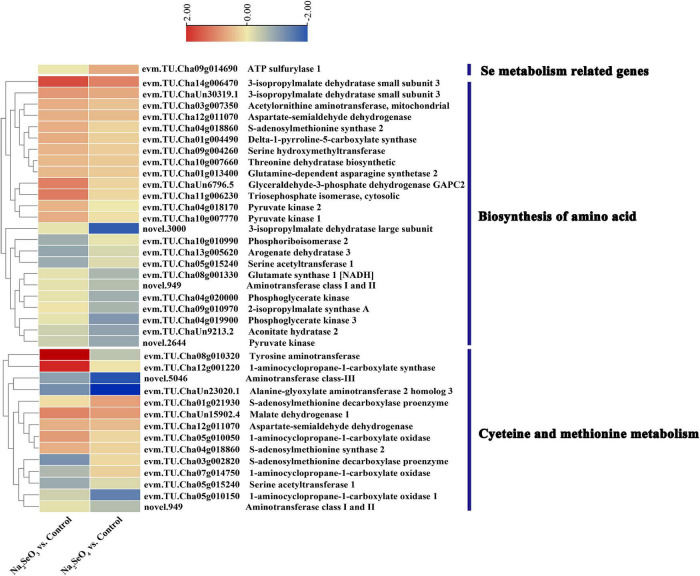
Heatmap of DEGs related to selenium and amino acid metabolism in response to selenite and selenate exposure.

Sulfate transporters mediate selenate uptake whereas phosphate transporters and aquaporin move selenite (White, 2016). However, only one sulfate and five phosphate transporter genes were identified for Na_2_SeO_3_ vs. control and Na_2_SeO_4_ vs. control, respectively, and no aquaporin gene was identified. We analyzed the expression patterns of all transporter-related genes to clarify their roles in response to selenite and selenate exposure in tea plants. For Na_2_SeO_4_ vs. control, the sulfate transporters evm.TU.Cha02g013540 (*SULTR1;1*) and evm.TU.Cha03g013130 (*SULTR2;1*) were upregulated 1.61- and 2.51-fold, respectively. For Na_2_SeO_3_ vs. control, the phosphate transporters evm.TU.Cha15g006520 (*PHT3;1a*), evm.TU.Cha09g013980 (*PHT3;1b*), and evm.TU.Cha09g000330 (*PHT1;8*) were upregulated 1. 53-, 1. 69-, and 1.51-fold, respectively. For Na_2_SeO_3_ vs. control, the aquaporins evm.TU.Cha01g023000 (*NIP2;1*) and evm.TU.Cha15g008600 (*NIP5;1*), were upregulated 5.95- and 1.53-fold, respectively (Supplementary Figure 2). For Na_2_SeO_4_ vs. control, only evm.TU.Cha09g014690 (ATP sulfurylase 1) was related to selenate reduction and its expression level was 1.59-fold higher in the selenate treatment than the control (Figure 5).

### Differentially Expressed Genes Involved in Amino Acid Biosynthesis and Cysteine and Methionine Metabolism

The GO enrichment and KEGG pathway analyses showed that the terms and pathways related to amino acid metabolism were highly enriched. Therefore, we analyzed the expression pattern of the DEGs related to amino acid biosynthesis and cysteine and methionine metabolism (Figure 5). For Na_2_SeO_3_ vs. control, 14 of 24 DEGs associated with amino acid biosynthesis were upregulated while evm.TU.Cha14g006470, evm.TU.Cha11g006230, and evm.TU.ChaUn6796.5 had increased by 3. 22-, 2. 25-, and 2.18-fold, respectively. For Na_2_SeO_4_ vs. control, 12 DEGs associated with amino acid biosynthesis were upregulated, evm.TU.Cha14g006470 expression had increased 2.17-fold, and 12 other genes were downregulated. For cysteine and methionine metabolism, evm.TU.Cha08g010320, evm.TU.Cha12g001220, and evm.TU.ChaUn15902.4 were upregulated 11. 31-, 4. 25-, and 2.11-fold, respectively, in response to selenite treatment. By contrast, no gene was upregulated by more than twofold in response to selenate treatment (Supplementary Table 6). Thus, the genes related to amino acid metabolism were more strongly induced by selenite than selenate.

### Differentially Expressed Genes and Differentially Expressed Proteins Involved in Glutathione Metabolism

**FIGURE 6:**
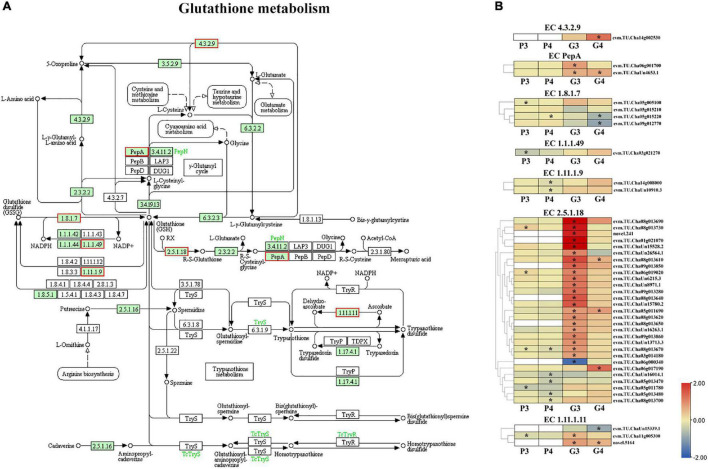
Genes and proteins involved in glutathione metabolism pathway. **(A)** Red rectangles represent DEGs or DEPs. **(B)** DEG and DEP heatmaps. P3 and P4 represent DEPs for Na_2_SeO_3_ vs. control and for Na_2_SeO_4_ vs. control, respectively. G3 and G4 represent DEGs for Na_2_SeO_3_ vs. control and for Na_2_SeO_4_ vs. control, respectively. Different colors indicate different levels of protein or gene expression based on log_2_ FoldChange. White rectangles indicate no DEGs or DEPs. Asterisks represent DEPs with *P* < 0.05 or DEGs with padj < 0.05.

A KEGG pathway analysis of the top 20 enrichments disclosed that the DEGs related to glutathione metabolism were enriched for both Na_2_SeO_3_ vs. control and Na_2_SeO_4_ vs. control. However, the DEPs related to glutathione metabolism were upregulated for Na_2_SeO_4_ vs. control (Figure 3). The combination of transcriptomic and proteomic data revealed 49 DEGs and their corresponding DEPs in the glutathione metabolism pathway (Figure 6). After selenite treatment, 29 DEGs of EC 2.5.1.8 (glutathione *S*-transferase, GST) and their DEPs were identified. Of these, 22 were upregulated. The genes evm.TU.Cha06g019020 and evm.TU.Cha08g013730 were upregulated at both the transcriptional and post-transcriptional levels. The genes evm.TU.Cha06g001700 and evm.TU.ChaUn4653.1 of EC PepA (leucyl aminopeptidase) as well as evm.TU.Cha11g005300 and novel.5164 of EC 1.11.1.11 (L-ascorbate peroxidase) were upregulated. Few genes responded to selenate fertilization (Supplementary Table 7). Hence, glutathione metabolism pathway was more strongly induced by selenite than selenate.

### Integrated Transcriptomic and Proteomic Dataset Analysis

**FIGURE 7:**
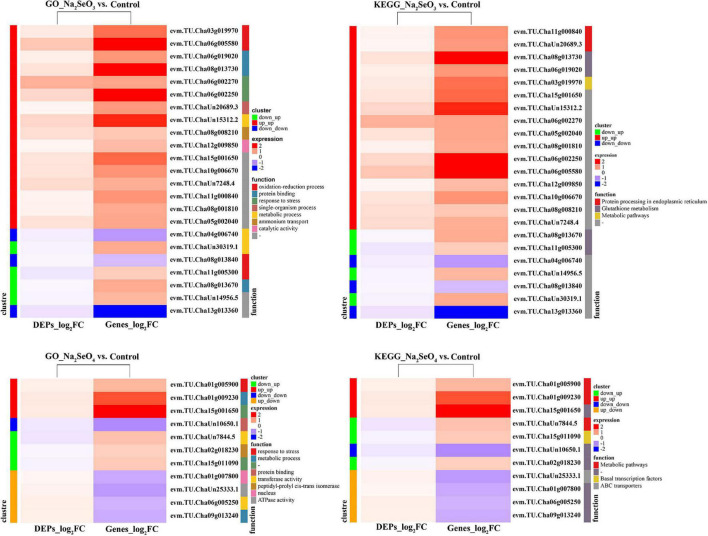
Heatmap of GO enrichment and KEGG pathway analyses of DEPs and their corresponding DEGs.

The GO enrichment and KEGG pathway analyses were conducted on integrated DEG and DEP data. For Na_2_SeO_3_ vs. control, the upregulated DEPs and DEGs were categorized under the GO terms ‘oxidation-reduction process,’ ‘protein binding,’ ‘response to stress,’ ‘single-organism process,’ ‘metabolic process,’ and ‘ammonium transport,’ and under the KEGG terms ‘protein processing in endoplasmic reticulum,’ ‘glutathione metabolism,’ and ‘metabolic pathways.’ For Na_2_SeO_4_ vs. control, the upregulated DEPs and DEGs were categorized under the GO terms ‘response to stress’ and ‘metabolic process’ and under the KEGG term ‘metabolic pathways’ (Figure 7).

### Differentially Expressed Genes Validation by RT-qPCR

**FIGURE 8:**
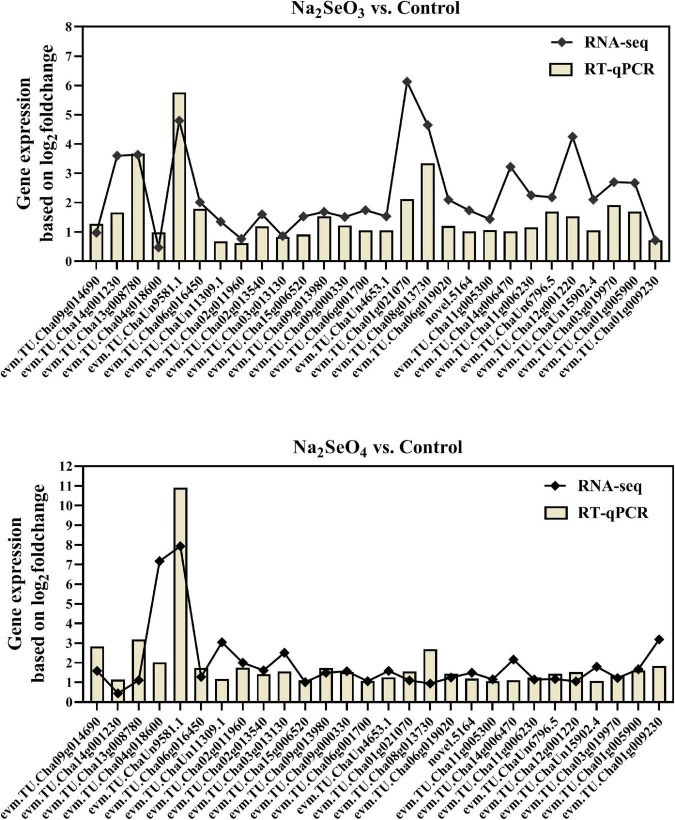
Validation of gene expression by RT-qPCR.

To verify RNA-Seq data accuracy and reliability, 28 genes related to the ABC, sulfate, and phosphate transporters, glutathione and amino acid metabolism, and others were selected for RT-qPCR analysis. The expression patterns demonstrated by RNA-Seq and RT-qPCR were consistent for 24 genes under Na_2_SeO_4_ vs. control and for 26 genes under Na_2_SeO_3_ vs. control (Figure 8). Therefore, the RNA-seq data were reliable.

## Discussion

Multi-omics technologies have been implemented in studies related to selenium disposition in many other plants. However, single omics method was usually applied toward this particular research in tea plants (Cao et al., 2018; Jia et al., 2020). In this study, both transcriptome and proteome techniques were applied to explore the transcriptional and post-transcriptional changes in tea plants after treated with selenite and selenate, respectively, illustrating the primary mechanism of selenium disposition in tea plants. Independent and joint analyses were carried out using the transcriptomic and proteomic data, and DEGs, DEPs and enrichment pathways were comprehensive discovered. In joint omics analysis, only a minimal fraction of DEPs and corresponding DEGs were identified, and the gene expression and protein expression also correlated modestly. Such observations are not unprecedented. Poor correlation between protein expression and gene expression changes were also commonly found in other species. We also speculated that the relative low-concentration of selenium of 5 μmol⋅L^–1^ and a short-time of 48 h treatment, which might not be sufficient to induce a large number of DEGs and DEPs, may account for this phenomenon. On the whole, the quality control and expression verification of omics data confirmed that our data are reliable, which will provide beneficial support for the study of selenium enrichment mechanism in tea plants.

### Se Uptake and Metabolism

After roots absorb selenite, they accumulate it and rapidly convert it to organoselenium compounds such as selenocysteine (SeCys), selenomethionine (SeMet), selenomethionine Se-oxide (SeOMet), and Se-methyl-selenocysteine (MeSeCys). By contrast, roots immediately translocate the selenate they absorb to the shoot (Li et al., 2008; White, 2016). Here, the highest total Se content was detected in the roots of plants supplemented with selenite and in the leaves of plants supplemented with selenate. Similar results were reported for maize and wheat (Li et al., 2008; Longchamp et al., 2015).

Integrated transcriptome and proteome analyses were conducted to clarify selenite and selenate uptake, translocation, and assimilation in tea plants. The ABC, nitrate, and sugar transporters were all responsive to selenite and selenate. The ABC transporter catalyzes ATP and provides energy for the transmembrane transport of substrates such as simple ions, peptides, complex lipids, and small proteins. The ABC transporter also plays important roles in biotic and abiotic stress response (Theodoulou and Kerr, 2015). In perennial ryegrass, ABCA transporters regulate Se movement and accumulation. *ATH* genes in the ABCA subfamily were upregulated in response to selenite exposure (Byrne et al., 2010). ABCC family genes control the disposition of cytotoxic and xenobiotic compounds and play crucial roles in stress tolerance (Kim et al., 2013). *ABCG14* participates in phytohormone transport (Gräfe and Schmitt, 2021). Selenite treatment upregulated the extracellular ABC transporter genes *ABCA2* and *ABCC4* while selenate exposure increased *ABCC8* and *ABCG14* expression. Therefore, the foregoing genes may also control Se uptake and tolerance in tea plants. Nitrate transporters regulate plant nitrate uptake and allocation. *NRT1;11* moves nitrate to young leaves. *NRT2;4* and *NRT2;5* regulate root nitrate absorption from the soil (Wang et al., 2018). Here, we found that selenite exposure induced *NRT2;4* and *NRT2;5* whereas *NRT1;11* and *NRT2;4* were upregulated by selenate in tea plants. NRTs may mediate both selenite uptake and selenate allocation. Sugar transporters mediate long-distance sucrose movement in the phloem (Julius et al., 2017). In this study, eight and six sugar transporter genes were upregulated in response to selenite and selenate treatment, respectively. Thus, sugar transporters might be implicated in selenite and selenate transport.

Sulfate transporters participate in selenate uptake while selenite absorption is mediated mainly by phosphate transporters and aquaporin. When tea plants were subjected to low-Se treatment (30 μmol⋅L^–1^ Se) for 4 days, 23 genes were downregulated and only *CsPHT3;1* was upregulated. When the tea plants were exposed to high Se levels (500–10,000 μmol⋅L^–1^ Se), most of their *CsPHT* genes were upregulated (Cao et al., 2021). Only a few DEGs, one sulfate transporter gene, and five phosphate transporter genes were identified in the present study possibly because the tea plants were exposed to only 5 μmol⋅L^–1^ Se. Therefore, we conducted other expression analyses on sulfate and phosphate transporters and aquaporin based on raw transcriptomic data. For the sulfate transporter, the high-affinity genes *SULTR1;1* and *SULTR1;2* are involved in selenate absorption (Rouached et al., 2008). The low-affinity genes *SULTR2;1* and *SULTR2;2* mediate sulfate transport from the roots to the leaves (Takahashi et al., 2000). We found that *CsSULTR1;1* and *CsSULTR2;1* were upregulated in response to selenate treatment. In tea plants exposed to selenate, Se accumulation was relatively greater in the leaves.

Most members of the PHT1 subfamily are associated with selenite and Pi uptake and translocation in plants (Song et al., 2017; Wang et al., 2017; Cao et al., 2021). PHT3 proteins play vital roles in Pi exchange between the cytoplasm and the mitochondrial matrix and are essential for ATP biosynthesis (Wang et al., 2017). Aquaporins are permeable to selenite and *OsNIP2;1* is related to selenite uptake (Zhao et al., 2010). In this study, *CsPHT1;3b*, *CsPHT1;8*, and *CsPHT3;1a* were upregulated in response to selenite treatment. Compared with the control, *CsNIP2;1* expression increased 5.95-fold following selenite exposure. For these reasons, *CsPHT1*, *CsPHT3*, and *CsNIP2;1* might play important roles in selenite uptake in tea plants.

Absorbed selenate utilizes the sulfur assimilation pathway, is reduced to adenosine 5′-phosphoselenate (APSe) and SeO_3_^2–^, and eventually forms organoselenium compounds (Guignardi and Schiavon, 2017). ATP sulfurylase (*APS*) is the first rate-limiting enzyme in selenate assimilation into APSe (Pilon-Smits and LeDuc, 2009). We found that *CsAPS1* was upregulated in the roots of tea plants subjected to selenate. Consequently, *CsAPS1* may play a vital role in selenate reduction.

Selenite assimilation occurs either enzymatically or non-enzymatically via glutathione (GSH). After selenite is absorbed by the roots, it is converted into organoselenium compounds (Pilon-Smits and Quinn, 2010; Guignardi and Schiavon, 2017). Here, we found that most genes related to glutathione metabolism were highly upregulated in the roots of tea plants treated with selenite. Hence, genes related to glutathione metabolism may be involved in selenite assimilation in tea roots.

### Responses of Amino Acid Metabolism-Related Genes to Selenite and Selenate

Selenite and selenate application for 4 weeks can significantly enhance the total amino acid content in tea plants (Hu et al., 2003). Here, selenite supplementation highly enriched GO terms related to amino acids, peptides, and proteins metabolism. Certain genes related to amino acid metabolism were differentially expressed in response to selenite and selenate treatment. Glyceraldehyde-3-phosphate dehydrogenase (GAPDH) induces protein expression and DNA repair (White and Garcin, 2017). Triose phosphate isomerase (TPI) plays an important role in the tricarboxylic acid (TCA) cycle and is indispensable in energy production (Lone et al., 2018). ACC synthase (1-aminocyclopropane-1-carboxylate synthase) catalyzes the biosynthesis of ethylene which plays a crucial role in plant tolerance to biotic and abiotic stress (Houben and Van de Poel, 2019). Malate dehydrogenase (MDH) oxidize oxaloacetic acid to malate and also enhances plant stress tolerance (Wu et al., 2007). Tyrosine aminotransferase (TAT) catalyzes the biosynthesis of the free radical scavenger vitamin E (Sandorf and Holländer-Czytko, 2002). Here, the foregoing genes were upregulated by over twofold in response to selenite fertilization but by not more than twofold following selenate supplementation. In tea plants, selenite treatment promoted amino acid and protein biosynthesis and enhanced stress tolerance in tea plants to a greater extent than selenate fertilization.

### Redox and Antistress-Related Gene and Protein Expression in Response to Selenite and Selenate

High Se concentrations are phytotoxic as they cause non-specific, disruptive selenoamino acid incorporation into proteins (Guignardi and Schiavon, 2017). Genes and proteins related to glutathione metabolism play important roles in assimilation and tolerance of Se in plants (Chauhan et al., 2020; Rao et al., 2021). After long-term selenite treatment on tea seedlings, glutathione metabolism was differentially regulated (Cao et al., 2018), remarkably, the expression of glutathione metabolism related genes and proteins were highly induced even with the short-term treatments of selenate and selenite in this study. Particularly, the antioxidant enzyme L-ascorbate peroxidase (APX) gene, related to glutathione metabolism and playing roles in protecting plants against oxidative stress (Uarrota et al., 2016), was significantly upregulated in the both treatments. Moreover, the importance of glutathione metabolism in tea plant response to selenate and selenite may not be the same. In response to selenite treatment here, 22/29 *GSTs* were upregulated in the roots and two of these genes were upregulated at both the transcriptional and post-transcriptional levels. By contrast, only three *GST* genes were upregulated in tea roots exposed to selenate. A GO enrichment analysis integrating transcriptome and proteome data showed that the two genes responding to selenite stress were upregulated at both the transcriptional and post-transcriptional levels. Nevertheless, only a single gene responding to selenate treatment was upregulated at both the transcriptional and post-transcriptional levels. The positive roles of GST in enhancing stress tolerance are highlighted in plants (Brentner et al., 2008; Xu et al., 2016; Wang M. et al., 2019). These findings suggest that for tea plants, selenite is relatively more phytotoxic than selenate and GST-mediated metabolism may be essential for tea plant detoxification. Definitely, further functional studies on the redox and antistress-related genes in the absorption and metabolism of selenium in tea plants should be undertaken in future.

## Conclusion

The present study combined transcriptome and proteome analyses to elucidate selenite and selenate uptake, allocation, and metabolism in tea plants. The Se content significantly increased in the roots of tea plants supplemented with selenite but significantly increased in the leaves of tea plants supplemented with selenate. The selenite treatment induced genes regulating the ABC, nitrate, and sugar transporters. The putative selenite uptake- and transport-regulating genes *PHT3;1a*, *PHT1;3b*, *PHT1;8*, and *NIP2;1* were also upregulated in tea plants subjected to selenite. Most genes and certain proteins associated with amino acid and glutathione metabolism and stress response were upregulated and may mediate Se assimilation and tolerance in tea plants. The selenate treatment induced genes regulating the ABC, nitrate, and sugar transporters as well as *SULTR1;1* and *SULTR2;1.* The latter two may participate in selenate absorption and translocation. Selenate exposure also induced ATP sulfurylase 1 which is the first rate-limiting step in selenate assimilation into APSe. The integrated analyses of Se content, genes, and proteins in this study may help clarify the mechanisms of selenite and selenate absorption and metabolism in tea plants.

## Data Availability Statement

The datasets presented in this study can be found in online repositories. The names of the repository/repositories and accession number(s) can be found below: National Center for Biotechnology Information (NCBI) BioProject database under accession number PRJNA795019. The mass spectrometry proteomics data deposited to the ProteomeXchange Consortium (http://proteomecentral.proteomexchange.org) have been permitted and assigned the dataset identifier PXD030944.

## Author Contributions

XH and JZ conceived and supervised the experiments. HR, XL, and LG performed the experiments. HR, XL, XH, and LW analyzed the results. HR wrote the manuscript. XH and JZ reviewed and edited the manuscript. All authors contributed to the article and approved the submitted version.

## Conflict of Interest

The authors declare that the research was conducted in the absence of any commercial or financial relationships that could be construed as a potential conflict of interest.

## Publisher’s Note

All claims expressed in this article are solely those of the authors and do not necessarily represent those of their affiliated organizations, or those of the publisher, the editors and the reviewers. Any product that may be evaluated in this article, or claim that may be made by its manufacturer, is not guaranteed or endorsed by the publisher.

## Footnotes

1
http://daehwankimlab.github.io/hisat2/

2
https://ccb.jhu.edu/software/stringtie/

3
http://subread.sourceforge.net/featureCounts.html

4
https://bioconductor.org/packages/release/bioc/html/DESeq2.html

5
https://pfam.xfam.org/search

6
http://130.88.97.239/PRINTS/index.php

7
http://prodom.prabi.fr/prodom/current/html/home.php

8
smart.embl-heidelberg.de

9
https://prosite.expasy.org

10
pantherdb.org

11
https://github.com/CJ-Chen/TBtools

12
https://softmany.com/adobe-photoshop-cs5/

## Supplementary Material

Supplementary Figure 1 Gene ontology (GO) enrichment analysis of differentially expressed genes (DEGs) in response to selenite and selenate treatment in tea plants.

Supplementary Figure 2 Heatmap of transcriptomic data related to putative selenite and selenate transporters. Different colors indicate different gene expression levels based on log2 FoldChange.
