# Supplementary figures and images for: Integrative Transcriptome and Proteome Analysis Reveals the Absorption and Metabolism of Selenium in Tea Plants [Camellia sinensis (L.) O. Kuntze]
Source: Front Plant Sci. 2022 Feb 24;13:848349. doi: 10.3389/fpls.2022.848349 (PMC8908381; doi:10.3389/fpls.2022.848349)

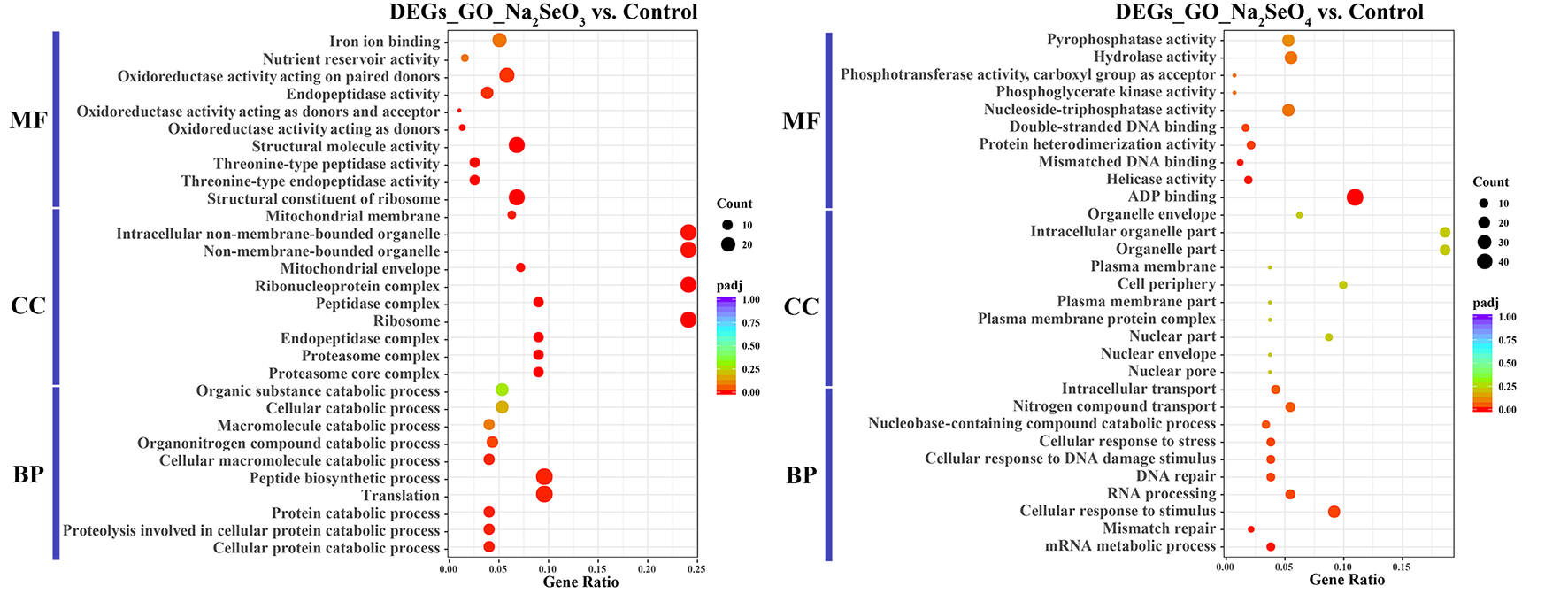

Supplement: Supplementary Figure 1 — Gene ontology (GO) enrichment analysis of differentially expressed genes (DEGs) in response to selenite and selenate treatment in tea plants. [file Image_1.TIF]

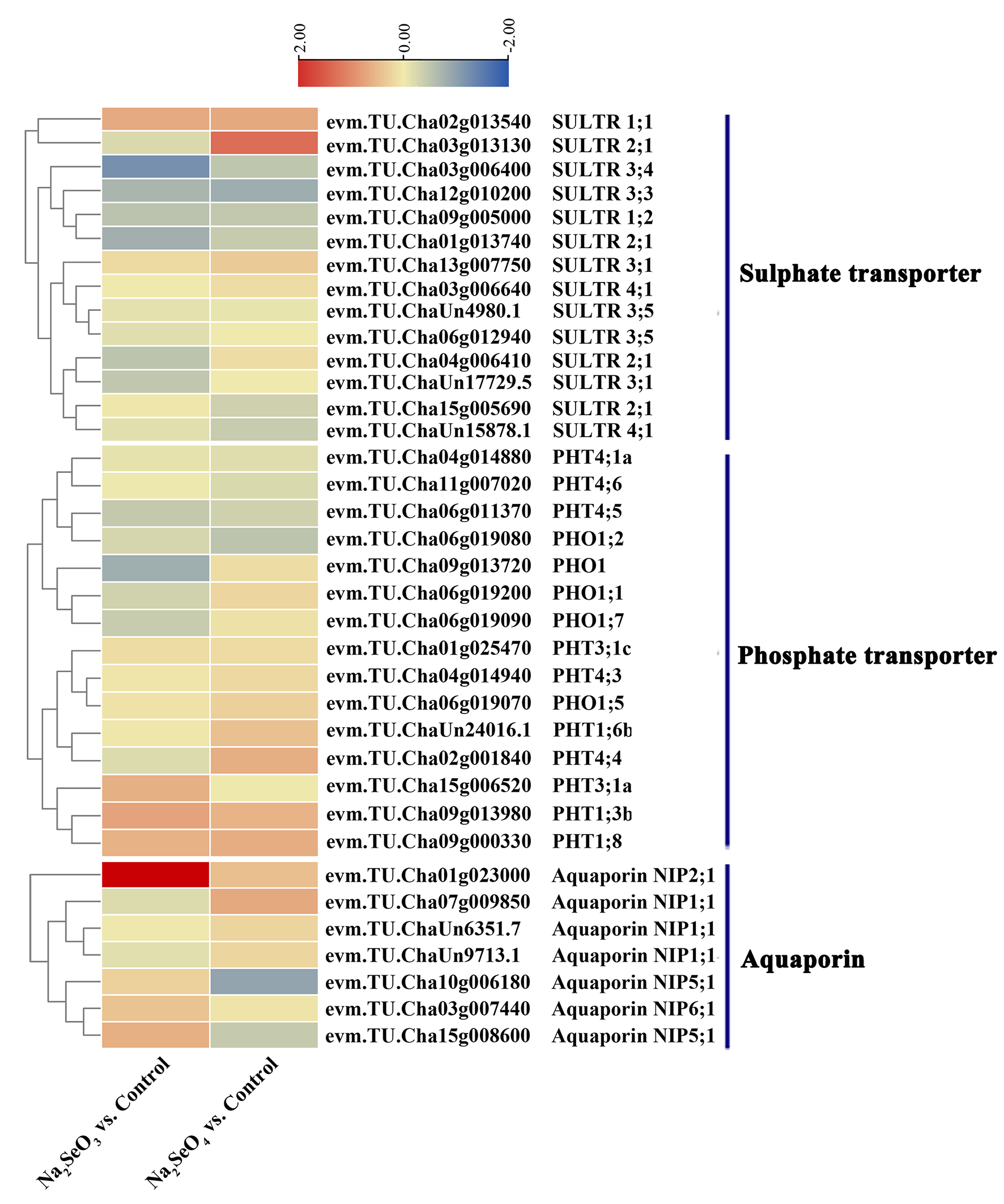

Supplement: Supplementary Figure 2 — Heatmap of transcriptomic data related to putative selenite and selenate transporters. Different colors indicate different gene expression levels based on log2 FoldChange. [file Image_2.TIF]
